# The impact of the Hamas-Israel conflict on the U.S. defense industry stock market return

**DOI:** 10.1371/journal.pone.0314677

**Published:** 2025-02-04

**Authors:** Jeroen Klomp

**Affiliations:** 1 Faculty of Military Sciences, Netherlands Defence Academy, Breda, The Netherlands; 2 Economics Section, Wageningen University and Research, Wageningen, The Netherlands; Obafemi Awolowo University, NIGERIA

## Abstract

On October 7th, 2023, Hamas carried out a terrorist attack on Israel. The Israeli government promptly retaliated the attack with massive bombing and a major military operation in the Gaza Strip. Following these events, a period of uncertainty started about the potential escalation of the internal conflict into a widespread regional conflict, or at least causes a threat to the fragile peace in the Middle East. This uncertainty was even further exacerbated as many regional leaders strongly condemned the actions of Israel. This study investigates whether the equity value of U.S. defense companies stands to gain in the short run from the risk of regional peace instability, given the anticipated increase in demand for arms. For this purpose, we rely on different approaches to capture the threat to regional peace caused by the internal conflict, including one based on the intensification of internet searches containing information about the Hamas-Israeli conflict. The key findings of this study unequivocally reveal that there is a significant positive relationship between the conflict intensity and the daily stock market returns of U.S. arms-producing firms. This result may suggest that investors are inclined to believe that the internal conflict is a serious threat to the regional peace, thereby enhancing the business prospects of the U.S. defense industry, as the largest arms supplier in the area.

## 1. Introduction

In the early hours of October 7^th^, 2023, Hamas carried out a surprise attack on Israel by launching thousands of Qassam rockets. At the same time, numerous Hamas terrorists managed to breach the border at multiple locations using explosive devices and infiltrated Israeli territory using armored vehicles and paragliders. This well-coordinated offensive resulted in the tragic deaths of at least 1,400 Israelis, most of them civilians, including 260 people at a music festival in Re’im. Besides that, hundreds of civilian hostages, including women, children, and elderly, were abducted and taken to the Gaza Strip. Hamas launched its attacks during the end of the Sukkot Jewish holiday, when many Israeli officers and soldiers were on leave. The assault marked the start of the most significant military escalation in the region since the Yom Kippur War exactly fifty years before and was the bloodiest in Israel’s history since the Holocaust (see [1, 2]).

After clearing Hamas militants, Israeli Prime Minister Benjamin Netanyahu declared during a television broadcast that Israel was at war. The Israeli military retaliated the attack by launching an extensive aerial bombardment campaign on Gazan targets, followed by a large-scale ground invasion of Gaza. Over the first six days of “Operation Swords of Iron,” more than 6,000 bombs were dropped on Gazan targets, causing an unprecedented and unparalleled civilian death toll. Israel also imposed a total blockade of the Gaza Strip, exacerbating a severe humanitarian crisis. The blockade led to critical shortages of water, electricity, fuel, food, and medical supplies. Based on unverifiable, and possibly inflated, figures reported by the Hamas government, in the first three months, more than 21,000 Palestinians, including over 8,000 children, lost their lives in the conflict (see [1, 2]).

In the weeks following the attack, many world leaders expressed their concerns that the intensifying violence between Israel, Hamas, and also Lebanon’s Hezbollah, would escalate into a large regional violent conflict [1]. The Middle East has been plagued by geopolitical tensions and conflicts for decades, driving the demand for advanced military equipment and technology to enhance defense capabilities. The region is the most important destination for U.S. arms exports. About forty percent of the total U.S. arms exports are directed to this region, with Saudi Arabia, the United Arab Emirates (UAE), and Israel as the major recipients. The United States maintains longstanding strategic alliances in the Middle East, often involving military cooperation and arms sales, aimed at countering some major regional military powers such as Iran. Additionally, the U.S. actively engages in counterterrorism efforts in the Middle East to combat common security threats, such as extremist groups [3, 4].

Given the unforeseeable nature of the Hamas attack and Israel’s response, it offers us a unique quasi-natural experimental setting to analyze the impact of unexpected armed violence on the expected profitability of the U.S. defense industry and test the efficient market hypothesis. Under the semi-strong form of the efficient market hypothesis, equity prices are assumed to reflect all public information and to adjust swiftly to the arrival of new public information (see [5–8]). Thus, when news related to the development of the Israel-Hamas conflict contains new or unexpected information that has important implications for the stability in the Middle East, the equity price of U.S. arms exporting companies will react immediately as it will alter market beliefs about arms trade conditions in the near future with this region.

Earlier studies on the impact of violent conflict on the stock market return of defense companies are foremost based on terrorist attacks or military operations (see, e.g., [9, 10]). Unlike unexpected terrorist attacks, military interventions are the final step of the conflict escalation ladder that usually starts with a diplomatic dispute between countries. Therefore, speculations about military actions typically take place already months or even years before the actual operation happens [11]. In that case, investors can better prepare themselves before the escalation. However, the most recent escalation between Israel and Hamas combines the sudden nature associated with terrorist attacks with the large-scale involvement of a military intervention. Additionally, this article contributes by noting that the recent eruption of violence between Hamas and Israel is the largest in decades, has caused an unparalleled humanitarian crisis, and has received unprecedented media and internet coverage. These issues have increased the awareness and attention of investors and may therefore affect their investment decision.

Thus, this study aims to explore the response of investors in the U.S. defense industry to the regional peace threat after the Hamas attack. For this purpose, we rely on three approaches to capture the threat to regional peace caused by the internal conflict. The first approach is based on the existence of a structural break after the attack in the stock market return of U.S. defense companies. The second approach involves the creation of a peace threat indicator based on the intensification of internet searches containing information about the conflict. Finally, we use the official casualty figures as an indicator of a further peace threat. In addition, as our dependent variable, we use the daily return on three U.S. defense industry-related exchange-traded funds (ETFs) between June 2023 and mid-March 2024. An ETF is an investment fund that tracks a specific index or fixed portfolio of stocks representing a particular industry or sector.

Our main findings reveal a significant positive relationship between the different conflict intensity indicators and the daily stock market return of U.S. defense firms. This result holds to several robustness checks, and we rule out any potential endogeneity concern by using an instrumental variables approach. In particular, the conflict caused a significant increase in the equity return of the U.S. defense industry by about ten percent. It appears that investors believe that the conflict is likely to be a real threat of the stability in the region, thereby enhancing the business prospects of the U.S. defense industry.

The remainder of the paper is structured as follows. The next section presents our theoretical considerations underlying the tested relationship. In section three, we provide our research design, while in section four, we report our estimation results. Finally, we end in section five with our conclusion and discussion.

## 2. Theoretical background

Earlier studies in economics and finance provide substantial empirical evidence that stock markets are prone to geopolitical risks, including wars, terrorist attacks, and violent civil protests (e.g., [9–21]). In economic sciences, terrorism refers the deliberate use of violence or threats by non-state actors to achieve political, ideological, or economic goals, often by disrupting economic stability, instilling fear, and causing direct and indirect economic losses [22]. Anticipating geopolitical risk demands forward-looking behavior since geopolitical risks not only compromise the likelihood associated with the actual occurrence of certain events, but also include the emergence of new risks resulting from escalating ongoing events [23]. The primary rationale underpinning the impact of geopolitical risks on stock markets is the uncertainty they create regarding the distribution of the economic costs. Specifically, geopolitical events tend to serve as a learning mechanism for investors, urging them to re-evaluate the risk components in their portfolios. In the aftermath of a sudden geopolitical shock, investors are likely to become more risk-averse and, as a reaction, try to escape into safer financial assets. They will continue to reshuffle their portfolios until the uncertainty related to the geopolitical events has disappeared again [24–27].

Over the past decade, numerous studies have delved into the impact of the ongoing internal Israeli conflict on the stock market (see, e.g., [28–31]). A prevailing consensus among these studies is that these events adversely affect the stock market returns in Israel and other Middle Eastern countries by heightening political and economic uncertainty [32, 33]. For instance, Kollias et al. [14] scrutinized the reaction of stock and bond indices of the Tel Aviv Stock Exchange before and during the military offensive that was launched against the Gaza Strip in late 2008. Albeit generally mixed, they revealed a distinct behavior among investors in these indices. Notably, the bond index exhibited significant positive abnormal returns during the conflict, indicating the preference of investors for the more stable bond market. However, as the military operations unfolded, investors apparently reverted to stocks. Plausibly, this behavior stems from anticipating a successful outcome of the offensive, considering the vast difference in military strength and the determination of the Israeli government.

However, the stock market return of defense-related companies might follow the opposite pattern compared to other industries. Specifically, the equity returns of non-defense-related firms are primarily affected by the economic uncertainty stemming from geopolitical events. In turn, defense-related stocks are much more influenced by the expected surge in demand for military items by countries who are highly susceptible to geopolitical risks, as well as, by those nations that plan to undertake military action against these risks (see i.e., [34–39]). Thus, while violent events may temper the economic activities of most business sectors, they may, at the same time, improve the economic outlook of defense companies [25, 40, 41]. The unstable climate originating from geopolitical incidents causes investors to expect increased dividends from these industries. For instance, the empirical results of Berrebi and Klor [40] reveal that while terrorist attacks negatively impact the equity returns of Israeli non-defense-related companies, they have a significantly positive effect on defense and security-related firms. Similarly, McDonald and Kendall [42] note that during periods of extreme violence, including armed conflicts and terrorist attacks, the U.S. defense sector experienced positive abnormal returns. Klomp [19] observes two opposing effects of the civil violence during the Arab Spring on the U.S. defense industry. In the short run, the stock market return of these countries increases due to the expected higher demand for military goods. Still, stock market prices fall in the long run when concerns about introducing arms embargoes become stronger and eventually dominate.

**Fig 1:**
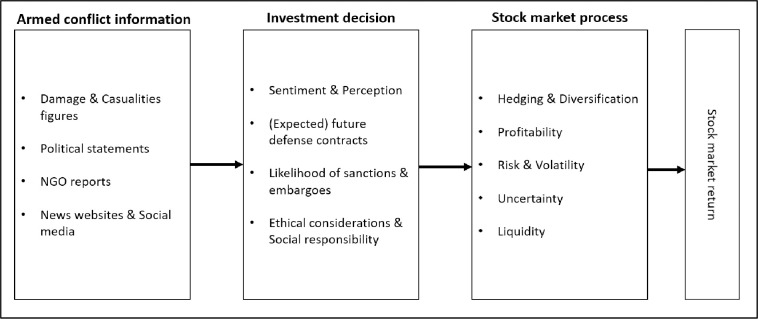
Chain of causality.

The blockchain diagram in Fig 1 formalizes the expected chain of causality of the impact of the latest Israel-Hamas conflict on the stock market performance of defense companies, thereby identifying different mediating channels. In the first stage, investors collect information about the severity of the conflict using different sources, including newspaper articles, casualty and damage figures reported by the Israeli government and Hamas, NGO reports on the human security situation, and political statements by Israeli politicians or foreign leaders. Investors also actively collect information about the conflict on the internet, mainly through search queries, news websites, and social media. In the second stage, potential investors process this information and initiate or adjust their investment decisions. The decision is mainly based on the economic opportunities induced by the conflict. For instance, in the first three months, the U.S. government supplied Israel with over 230 fighter jets and 20 navy vessels. However, the complete demand effect might go beyond the direct increase in arms demand by Israel. In particular, investors might expect that the Israel-Hamas conflict will escalate into a widespread regional conflict in the Middle East. This expectation is likely to be further exacerbated after the hostile responses from Iran and Hezbollah, along with the bombing of military positions in Lebanon and Syria by Israel and the U.S. Many countries in the Middle East, predominantly those with Arab populations, have historically expressed solidarity with the Palestinian cause, condemning Israeli actions. This support manifests in many different ways, including diplomatic statements, protests, military support, and financial aid. One important remark in this respect is that the Palestinian cause is clearly not equal to the interests of Hamas. The Palestinian cause broadly seeks the establishment of a sovereign state for Palestinians, advocating for human rights and self-determination. In contrast, Hamas, an Islamist militant organization designated as a terrorist group by many countries, pursues its own political and ideological goals, including the establishment of an Islamic state and the destruction of Israel. While some Palestinians support Hamas, others do not, indicating that Hamas’ interests do not represent the entirety of the Palestinian cause [43].

Several Western intelligence agencies have accused Iran and Hezbollah of actively assisting Hamas in the terrorist attack [44]. Other Middle East countries are more passively supporting Hamas. For instance, the Syrian foreign ministry called the Hamas operation an "honourable achievement that proves the only way for Palestinians to obtain their legitimate rights is resistance in all its forms". In a public statement, the Saudi Arabian Ministry of Foreign Affairs expressed their concerns that the unprecedented developments between various Palestinian factions and the Israeli armed forces would lead to an escalation of violence on several fronts in the region, or at least to a threat to the relative peace in the region [45].

Moreover, Iranian parliament members chanted “Death to Israel,” and crowds in Teheran burned Israeli and U.S. flags. Meanwhile, Houthi rebels in Yemen said in an announcement that they are supporting "the heroic jihadist operation," emphasizing “the revealed weakness of Israel.” In the months following the attack, Houthi rebels attacked and entered merchant ships sailing through the Red Sea under the flags of countries that publicly supported Israel. As a response, the U.S. and U.K. bombed Houthi facilities in Yemen. Moreover, Israel’s killing of a high-ranked military advisor of the Iranian Revolutionary Guard and the assassination of several Hamas leaders on Lebanese soil, led to Iran vowing retaliation [45].

Based on these hostile reactions, a further escalation of the conflict may not come as a surprise. One can, therefore, argue that arms suppliers to the Middle East region will benefit from this explosive situation, especially from the U.S., as the largest arms supplier in the region [46]. However, investment decisions are not always based only on economic arguments, but also on ethical considerations. For different reasons, investing in the defense industry is often also seen as unethical. First, the defense sector profits from conflict and war, which inherently involve human suffering, loss of life, and destruction. By investing in companies that manufacture weapons and military equipment, individuals indirectly contribute to the perpetuation of armed conflicts and violence, often at the expense of innocent civilians [47]. Second, the military-industrial complex can exert undue influence on government policies, leading to increased militarization and focusing on military solutions to international conflicts rather than diplomatic or peaceful resolutions [48]. Third, many arms-producing companies do not fulfil the criteria for corporate social responsibility and operate with limited transparency and accountability, raising concerns about corruption and human rights violations [49–51].

Meanwhile, when investors behave rationally, they will also consider that when the Hamas-Israel conflict becomes a threat to the peace in the region or even escalates into a wider conflict, it will increase the likelihood of more rigorous export control policies or could even lead to arms embargoes to particular countries or regions. This latter effect will hamper U.S. arms exports to the Middle East again or at least make it more expensive as compliance, transportation, financing, and brokering costs may rise [19]. For instance, the Dutch court has ordered the government of the Netherlands to stop exporting F-35 fighter jet parts as there is a clear risk that severe violations of humanitarian law of war are committed in the Gaza Strip with Israel’s F-35 fighter planes [52]. Yet, these parts were actually owned by the U.S. and only stored by the Netherlands in a regional warehouse. Nevertheless, the risk for more stringent arms trade policies, in the long run, might lead to a hoarding effect in the short run, thereby spurring current U.S. arms exports.

The hypothesis tested in this study is given by.

**H**_**1**_: *An increase in a regional peace threat caused by the intensification of the Israel-Hamas conflict raises the stock market return of U*.*S*. *defense companies*.

## 3. Research design

### 3.1 Data

One of the key challenges in analyzing the stock market impact of the Israel-Hamas conflict is finding a suitable indicator that captures the further escalation of the Israel-Hamas conflict and the threat to regional peace in the Middle East. In our attempt, we follow two approaches. First, we create a dummy variable taking the value one on the first *n*-trading day of the U.S. stock market after the terrorist attack by Hamas. This variable allows us to investigate whether there is a structural break in the defense stock market return following the onset of the recent conflict and to assess the duration of this break by comparing different time windows. When the conflict intensifies, the persistence of the structural break is likely to become more significant.

However, one major drawback of this approach is that the dummy variable does not take into account how changes in the conflict intensity influence the stock market return. To address this limitation directly, we follow the methodology proposed by Dreger et al. [53] and Hoffmann and Neuenkirch [54]. This approach builds on the assumption that when a conflict intensifies and becomes more violent (i.e., creates more civil casualties or large-scale destruction), it will be more widely covered in newspaper articles, the media, or receive more attention on the internet, specifically on social media platforms or news websites [19]. In particular, investors update their expectations about the economy more frequently during periods of high news coverage than during periods of low news activities [55]. To measure this, we utilize a daily risk indicator based on internet search volume data collected by Google. Unlike traditional methods relying on news articles published in international newspapers, this approach provides a more direct proxy for investor attention, as users actively search for relevant information if they are interested in the underlying matter (see i.e., [56–59]).

The search volume for a specific keyword provided by Google Insights is not given in absolute terms, but as a value relative to the total number of Google searches in a predefined time interval. For each search term, this relative value is then normalized so that the search volume continuously varies between 100 (i.e., a period in which the highest relative volume was observed) and zero (i.e., a period in which search volume does not meet a designated threshold). In our attempt, we consider all search queries containing a joint occurrence of the keyword “Israel” in any combination with “Hamas,” “Israel Defence Force,” “Hezbollah,” “Operation Iron Sword,” “Qassam Brigade,” “Al Shifa” (the battle for the Al Shifa hospital was one of the fiercest during the ground offensive of Gaza), “Khan Younis” (a refugee camp that was heavily under attack) or “Gaza”. The basic idea behind this approach is based on the assumption that when the risk of the internal conflict spreading in the region escalates, it will be more extensively covered in internet search queries by investors seeking information. Increased attention to the conflict illustrates the uncertainty of stock market investors regarding how to perceive the risk of further escalation and the threat to regional peace. In essence, low-risk events will be less covered in search queries as investors might care less about these events, as they have only a limited impact on the stock market return of their portfolio. Thus, investors—in advance of any actual regional escalation—rearrange their portfolios based on their own assessments of the likelihood of escalation. In turn, this risk assessment is built on (i) the severity of the underlying conflict in the Gaza Strip, (ii) the threats expressed by several non-state actors and, (iii) the responses by state leaders in the Middle East and the rest of the world.

**Fig 2:**
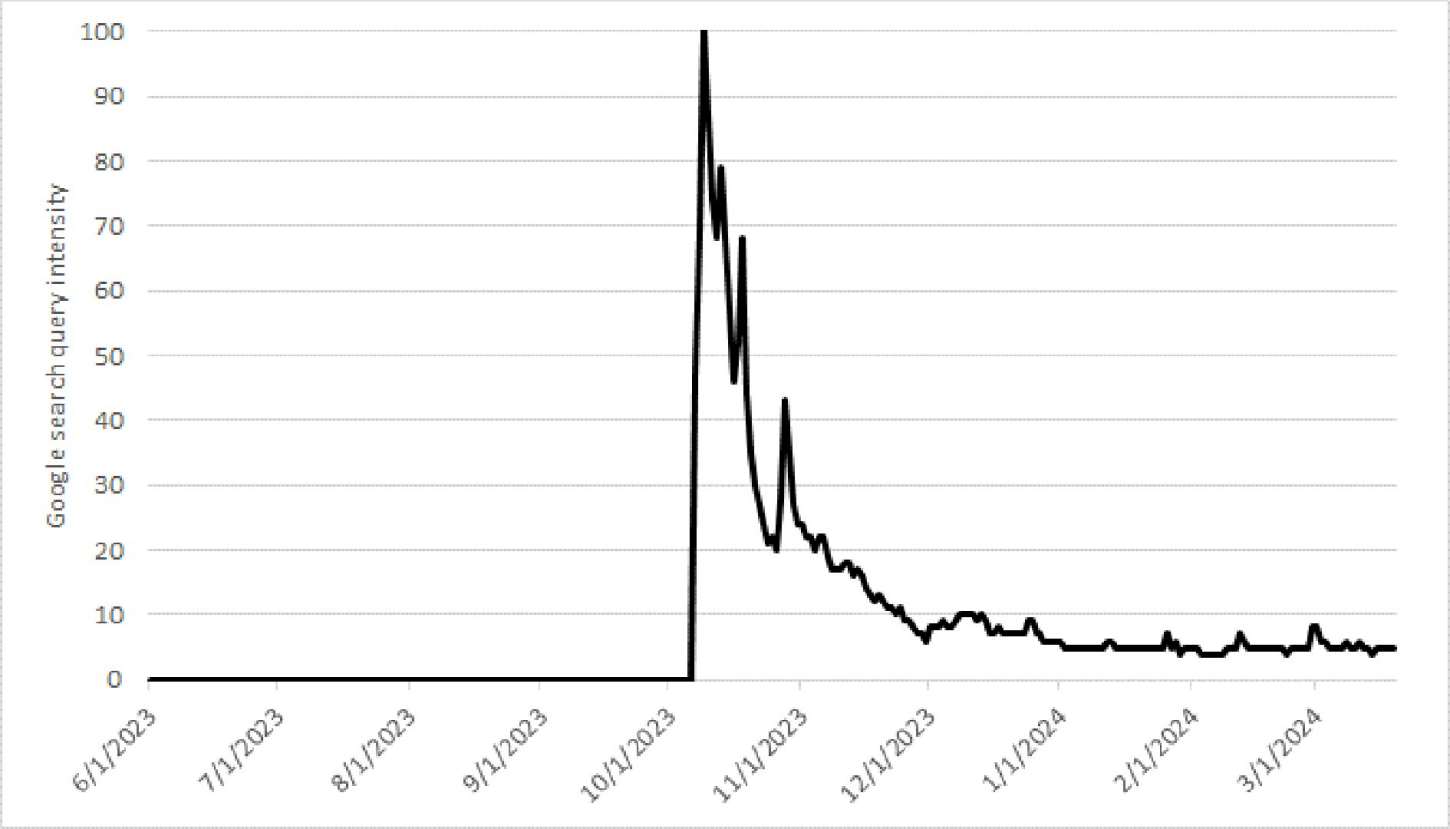
Google search queries.

We compute the search density for each day for a joint occurrence of the keywords between June 1^st^, 2023, and March 19^th^, 2024. Since the combinations of search terms do not fully guarantee that a particular news item contains information about the Hamas attack, the military response by Israel, the likelihood of escalation, or the threat to regional peace, we set the composite news index to zero from June 1st, 2023, through October 6th, 2023 (see also [53]). Fig 2 shows the density of conflict-related Google searches during the period of analysis (y‐axis). The risk indicator immediately jumps after the date of the Hamas attack, but diminishes rather quickly in the weeks thereafter. Based on this data, we create our risk variable by computing a cumulative indicator on the relative number of searches. We apply a log plus one transformation to the indicator variable as the information content of an individual search diminishes with the total number [55]. To proxy the true news component from an investor perspective, we include the first-difference of this transformed indicator as the risk variable in our econometric model later on.

**Fig 3:**
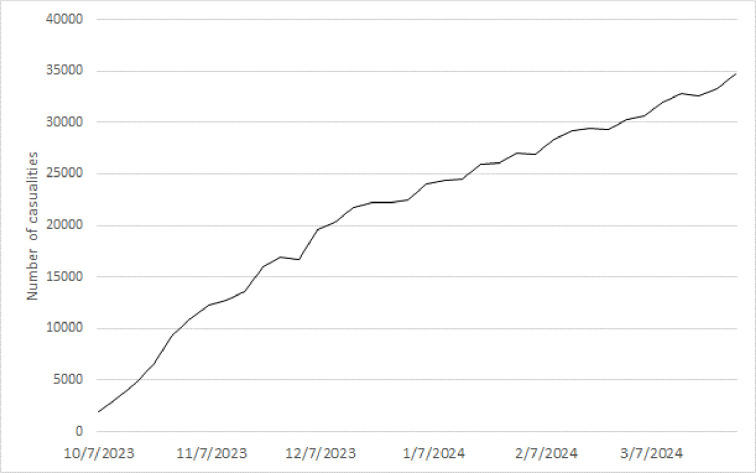
Casualties in Israel and Gaza.

Finally, as an alternative indicator of the conflict’s intensity, we use the daily rate of change in the accumulated number of casualties (combining both Israeli and Palestinian casualties) reported by the Hamas-led Gaza Health Ministry and the Israeli Ministry of Interior (see Fig 3). As already argued above, when the number of casualties increases, the public will more frequently gather information about the event as it might be expected that it will escalate in the near future. However, it’s crucial to approach these figures cautiously due to their lack of verifiability and susceptibility to measurement errors or biases. In particular, Hamas has strong incentives to inflate statistics and the opposite holds for the IDF. Both sides have been shown to report incorrect numbers on specific incidents (see e.g., [60]).

To measure the market value of the defense industry in the United States, we use exchange-traded fund (ETF) data (see also [61]). An ETF is a portfolio of shares that tracks the yield and return of a sector-specific portfolio of stocks. It is publicly traded like any other company stock on the stock exchange. ETFs have become one of the most popular investment tools among investors, because of their tax efficiency, low transaction costs, high transparency, and high intraday liquidity [62, 63]. We use pricing data from three daily traded U.S. Aerospace and Defense ETFs (iShares, Standard & Poor’s, and Invesco) from June 2023 to mid-March 2024. These ETFs are good representatives of the U.S. defense industry as the companies included in the ETFs cover a large majority of the total revenues received by U.S. defense companies. Table A1 in the S1 Appendix reports the top ten holdings for each ETF, while Table 1 reports the summary statistics for the daily return series for the considered ETFs. It turns out that the Jarque–Bera test rejects the null hypothesis of normality for most series. The distribution of the ETF returns exhibits positive skewness and excess kurtosis.

**Table 1:** Descriptive statistics.

	Observations	Mean	Standard deviation	ADF	Pr(Skewness)	Pr(Kurtosis)	Pr(Joint normality)
	(1)	(2)	(3)	(4)	(5)	(6)	(7)
iShares	201	0.0008	0.0091	0.037	0.012	0.001	0.001
S&P	201	0.0011	0.0107	0.017	0.031	0.011	0.027
Invesco	201	0.0012	0.0078	0.038	0.041	0.035	0.014

This study is based on the efficient market hypothesis. One crucial assumption underlying this hypothesis is that stock prices follow a random walk pattern, meaning that future price movements cannot be predicted based on past prices. Thus, under the efficient market hypothesis, stock returns are assumed to exhibit a stationary behavior. The augmented Dickey-Fuller (ADF) test is used to test the (non-)stationarity of each price series [64]. The null hypothesis of this test is that all series are non-stationary. The lags included in the ADF regressions are selected based on the Akaike Information Criterion (AIC). The results in column (4) of Table 1 suggest to accept the null of unit roots for most of the ETF price series in the level form at the five percent significance level, whereas the variables are mainly stationary in the first-difference form.

**Fig 4:**
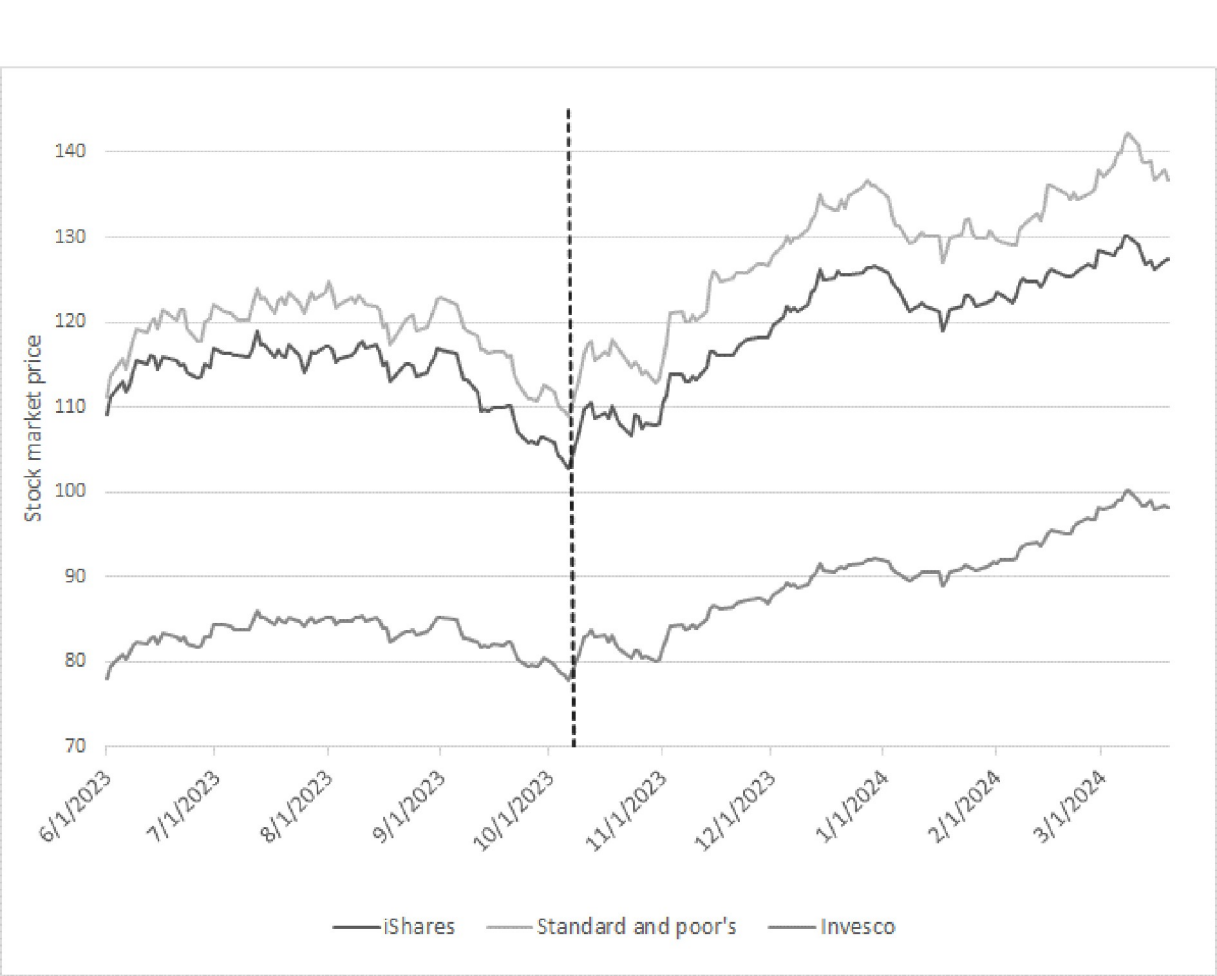
Price development of Aerospace & Defence ETFs.

In Fig 4, we report the price development of the different ETFs in our period of analysis. The pairwise correlation among the three ETFs is high and ranges between 0.85 and 0.92. As a preliminary test, we compare the stock market price of the U.S. defense companies before and after the Hamas attack. Based on the graph, there is a clear increase visible in the stock prices in the considered ETFs in the days after the Hamas attack. Simple t-tests on this structural break indicate that the returns are significantly higher at common statistical confidence levels after the event. However, these nonparametric tests are only suggestive as other confounding variables are not taken into account.

### 3.2 Empirical model

This section presents the empirical model used to estimate the impact of the violent conflict between Israel and Hamas, and its contagious effect on the sustainability of peace in the region, on the return of three U.S. defense-related ETFs. One concern is that the time series used for this return exhibits time-variation and clustering. For this reason, an EGARCH specification is used, which jointly models returns and volatility. This model corrects for the kurtosis, skewness, and time‐varying volatility present in the equity return (see [65–67]). More formally, the general specification of the mean equation is given as follows.


$$r_{t}=\alpha +\beta r_{t-k}+\gamma x_{t-k}^{j}+\theta {conflict}_{t}+\delta_{day}+\delta_{month}+\varepsilon_{t}`$$ (1)

Where *r*_*t*_ is the return at day *t* of a specific U.S. Aerospace and Defense ETF (based on opening prices). The lagged return *r*_*t−k*_, is included to control for autoregressive tendencies and serial correlation. The vector **x**^*j*^ is our vector with control variables, including *j* elements. The optimal number of *k*-lags on the control variables is determined using the Schwarz-Bayesian Information Criterion (SBC). The variable *conflict* represents our proxies for the escalation risk or peace threat perceived by investors after the Hamas attack on Israel, as discussed in the previous section. The parameter *θ* can be interpreted as the abnormal return linked to the expectations of the Israel-Hamas conflict on regional peace. Our hypothesis posits that investors react to new information that could have important implications for the demand for arms in the Middle East region. When the conflict risk rises, this will reinforce a positive investor sentiment as it might trigger a regional arms race. Thus, we expect that *θ* > 0 in Eq (1).

In the vector of control variables, we consider variables suggested by previous studies explaining the stock market returns of defense companies and are required to avoid an omitted variable bias [24, 41]. First, we include the ten-year U.S. Treasury bond interest rate and three-month U.S. T-bill rate to control for the long and short-term interest rates. An increase in the interest rate will decrease the present value of the expected future cash flow. At the same time, a rise in the interest rate increases the debt burden for defense companies. To control for the market return and producer prices, we include respectively the return on the S&P500 and the monthly producer price index reported by the Federal Reserve.

Moreover, the Middle East is one of the most important regions for oil exports. The risk of a broader violent dispute might increase the uncertainty about the number of barrels of oil traded on the world market in the near future. This will lead to upward pressure on the oil price. In turn, this price uncertainty might create spillover effects and have important implications for monetary policy. For instance, investors might be concerned that the exchange rate will start to appreciate, reducing the competitiveness of the U.S. defense industry. So, there could be a danger that our results do not reflect the impact of the conflict risk or peace threat on the equity return of the U.S. defense industry, but instead, reflect the relationship between the particular ETFs and other financial assets or commodities. To address this issue, we include the daily Brent spot oil price and the real exchange rate in our analysis.

Meanwhile, since the invasion of Ukraine by Russia in February 2022, the Biden Administration has been supporting Ukraine in this battle. During the period of our analysis, the U.S. government granted Ukraine several arms packages. This commitment is expected to benefit the U.S. Defense industry as well. We include a dummy variable to control for this issue, taking the value one on the two consecutive days after Congress has approved a package. Additionally, we include the daily volume of trade of a specific ETF to capture its degree of liquidity. Finally, day-of-the-week and month-fixed effects are added to control for trading day effects.

Table 2 provides the descriptive statistics of the control variables used. According to this table, all control variables are stationary after their first-difference. This means that the null hypothesis of unit root (non-stationarity) is rejected. In the next step, we check for cointegration using the empirical tests suggested by Pedroni [68]. This test has a null hypothesis of no cointegration in the long run. The results reported in the final columns of Table 2 suggest that the null hypothesis of no co-integration can be rejected for most of the time series. Hence, it may imply that there is a long-run steady-state relationship between the variables included in our EGARCH model.

**Table 2:** Descriptive statistics control variables.

	Mean	Standard deviation	ADF test (p-value)				Phillips–Perron unit root test (p-value)			
			Level		First difference		Level		First difference	
	(1)	(2)	(3)		(4)		(5)		(6)	
3 months U.S. T-bill rate	0.052	0.001	0.373		0.034	**	0.461		0.027	**
10-year U.S. government bond interest rate	0.041	0.003	0.489		0.021	**	0.511		0.027	**
Return S&P 500	0.001	0.007	0.679		0.040	**	0.326		0.023	**
Change in the Producer Price Index	0.011	0.005	0.636		0.015	**	0.791		0.030	**
Change in the oil price	0.001	0.017	0.748		0.023	**	0.661		0.011	**
Change in real effective exchange rate	0.0001	0.003	0.603		0.036	**	0.471		0.043	**
Weapons deal Ukraine dummy	0.085	0.278	0.035	**	0.017	**	0.503		0.018	**
Trade volume iShares (in logs)	13.001	0.389	0.784		0.039	**	0.459		0.013	**
Trade volume S&P (in logs)	11.614	0.583	0.626		0.032	**	0.717		0.015	**
Trade volume Invesco (in logs)	11.181	0.497	0.779		0.010	**	0.364		0.044	**

Note

**/* Indicating significance levels of respectively 5 and 10 percent.

The model given in Eq (1) is estimated using the Generalized Error Distribution as the assumption of normally distributed residuals is violated. This approach nests the normal and several other densities. In particular, as conditional heteroskedasticity is only partially responsible for the leptokurtosis observed in the return series, standard errors obtained under the normality assumption tend to understate the true standard errors (see [65]). Given our relatively small sample of daily observations, we apply the bootstrap procedure with 1,000 replicators to obtain robust standard errors.

The conditional variance *h*_*t*_ can be expressed as a function of the lagged standardized innovations *ε*_t-1_/*h*_t-1_ and the lagged conditional variance *h*_t-1_. Therefore, the variance equation is given by.


$$ln(h_{t})=\vartheta +\eta_{1}\left|\frac{\varepsilon_{t-1}}{h_{t-1}}\right|+\eta_{2}\left(\frac{\varepsilon_{t-1}}{h_{t-1}}\right)+\eta_{3}ln(h_{t-1})$$ (2)

If *η*_*2*_ equals zero, there are symmetric effects, but if *η*_*2*_ is positive (negative), high (low) price news generates more volatility. The mean and variance equations are simultaneously estimated using Bollerslev and Wooldridge’s maximum likelihood estimator.

## 4. Results

Table 3 presents the estimation results of the EGARCH model using a dummy variable taking the value one on the first *n*-trading days in the U.S. after the Hamas attack. This dummy allows us to test if there is a structural break present in the ETF time series. To assess the persistence of any structural break, we evaluate the effect of the terror attack and the subsequent military operation using different time windows, i.e., [0,1], [0,3], [0,5], [0,10], and [0,30]. It appears that the onset of the conflict exerted upward pressure on the stock market return of U.S. defense companies, as the dummy variable for the post-Hamas attack is highly significant across the different time windows. It turns out that the effect increases over the time period, indicating that investors become more concerned as the conflict starts to unfold. Specifically, the ETF return increased by about 10 percentage-points within the thirty trading days after the Hamas attack.

**Table 3:** U.S. defense ETFs and Israel-Hamas conflict—Dummy variable.

	(1)		(2)		(3)		(4)		(5)	
Time window:	[0,1]		[0,3]		[0,5]		[0,10]		[0,30]	
	*iShares*									
Lagged dependent	0.310	**	0.344	**	0.280	**	0.315	**	0.281	**
	(0.112)		(0.139)		(0.132)		(0.083)		(0.120)	
Attack date dummy	0.061	**	0.095	**	0.103	**	0.120	*	0.138	*
	(0.024)		(0.037)		(0.042)		(0.071)		(0.084)	
Observations	196		196		196		196		196	
Pseudo R-squared	0.18		0.23		0.22		0.27		0.25	
Ljung-Box test (p-value)	0.26		0.39		0.37		0.32		0.32	
μ_1_	0.36		0.22		0.18		0.21		0.22	
μ_2_	0.24		0.10		0.14		0.30		0.24	
	*Invesco*									
Lagged dependent	0.329	**	0.274	**	0.287	**	0.274	**	0.283	**
	(0.120)		(0.101)		(0.100)		(0.137)		(0.080)	
Attack date dummy	0.052	**	0.055	**	0.060	**	0.066	*	0.074	*
	(0.022)		(0.027)		(0.028)		(0.034)		(0.039)	
Observations	196		196		196		196		196	
Pseudo R-squared	0.09		0.11		0.10		0.12		0.13	
Ljung-Box test (p-value)	0.29		0.24		0.27		0.26		0.32	
μ_1_	0.19		0.11		0.32		0.17		0.32	
μ_2_	0.19		0.32		0.14		0.15		0.20	
	*Standard & Poor’s*									
Lagged dependent	0.310	**	0.281	**	0.279	**	0.357	**	0.341	**
	(0.094)		(0.075)		(0.086)		(0.154)		(0.159)	
Attack date dummy	0.044	**	0.062	**	0.066	**	0.073	*	0.083	*
	(0.012)		(0.017)		(0.018)		(0.038)		(0.050)	
Observations	196		196		196		196		196	
Pseudo R-squared	0.18		0.26		0.20		0.27		0.20	
Ljung-Box test (p-value)	0.31		0.32		0.29		0.22		0.39	
μ_1_	0.24		0.11		0.37		0.25		0.13	
μ_2_	0.14		0.29		0.34		0.25		0.20	

Notes: Results of simultaneous estimation of Eqs (1) and (2) using maximum likelihood including the control variables outlined in the main text. Standard errors are heteroskedasticity consistent

**/* Indicating significance levels of respectively 5 and 10 percent.

However, as previously already mentioned, one major drawback of using a dummy variable is its failure to capture changes in the intensity of the conflict. To address this limitation, we make use of the Google search intensity data. We employ a rolling average window of two days for our search indicator since information may be released after the closing of the trading system. The estimation results in columns (1)-(3) of Table 4 indicate that an increase in the search intensity raises the return in all three ETFs at common statistical confidence levels across all specifications. This suggests that news searches about the conflict significantly fuel the speculations of its escalation, fostering a positive sentiment among investors in the U.S. defense industry who view the conflict as an opportunity to expand arms sales.

**Table 4:** U.S. defense ETFs and Israel-Hamas conflict–Intensity.

	iShares		Invesco		Standard & Poor’s		iShares		Invesco		Standard & Poor’s	
	(1)		(2)		(3)		(4)		(5)		(6)	
Lagged dependent	0.309	**	0.378	**	0.295	**	0.309	**	0.355	**	0.218	**
	(0.028)		(0.013)		(0.026)		(0.035)		(0.010)		(0.033)	
Google searches	0.034	**	0.029	**	0.024	**	0.021	**	0.021	**	0.018	**
	(0.009)		(0.009)		(0.031)		(0.044)		(0.047)		(0.044)	
Estimation method	GARCH						GARCH-IV					
Observations	196		196		196		196		196		196	
Pseudo R-squared	0.08		0.13		0.17		0.15		0.15		0.10	
Ljung-Box test (p-value)	0.22		0.40		0.40		0.27		0.24		0.34	
μ_1_	0.37		0.17		0.32		0.12		0.27		0.12	
μ_2_	0.28		0.32		0.39		0.29		0.35		0.24	
*First stage*												
Casualties									0.283	**		
									(0.029)			
Wald test first stage									297.24			

Notes: Results of simultaneous estimation of Eqs (1 or 4) and (2) using maximum likelihood including the control variables outlined in the main text. Standard errors are heteroskedasticity consistent

**/* Indicating significance levels of respectively 5 and 10 percent.

To provide an approximation of the overall impact of conflict-related search queries on the considered defense-related ETFs, we multiply the cumulative absolute changes of the uncertainty indicator by the coefficients found in the first part of Table 4. It appears that the internal conflict has raised the stock price by about 13 percent in the period of our analysis.

Moreover, it is essential to check the results for serial correlation among the error terms as violating this assumption may negatively affect our causal inference, for instance, due to endogeneity problems. However, the insignificance of the Ljung-Box tests provides no evidence of a serial correlation. Thus, we reject the null hypothesis of the Ljung-Box test and safely assume that the data is independently distributed.

In the regressions so far, we assumed that the Google search intensity is exogenous. This assumption is supported by the absence of any multicollinearity with the included covariates, as can be witnessed from the correlation matrix in Table A3 in the S1 Appendix. It appears that none of the pairwise correlations exceed the commonly used threshold of 0.8. Additionally, a reversed causality issue can be excluded as well, as it is hard to imagine that the internet search intensity about the conflict is driven by the expected profitability of U.S. arms-producing companies. Only a very small portion of the population that searches for information about this conflict is likely to own these particular stocks. However, it is possible that the stock market return of defense companies and the internet search intensity are driven by the same underlying factors. When we fail to control for these factors explicitly, our results might be spurious and suffer from endogeneity concerns. To address this endogeneity issue, we apply the Instrumental Variable (IV) estimation technique [69]. As our instrument, we use the total number of Google searches within our period of analysis. Theoretically, the search queries on which the risk indicator is based are not at the top of the ranking on the most popular search topics during our complete time frame. Therefore, it is likely that the total number of Google searches will not directly affect the stock market return of a selective number of companies. At the same time, the peaks in the total number of searches are around the first days of the conflict. Thus, our instrument is likely to be correlated with our measure on the likelihood of escalation of the conflict and not directly with our dependent variables. This latter is also confirmed by the low pairwise correlations between the daily stock market return of the defense companies and the daily total number of search queries. More specifically, the first-stage regression is estimated using the OLS estimator and is provided as follows.


$${conflict}_{t}=\omega +\varphi \;\ln\;{searches}_{t}+\vartheta_{day}+\vartheta_{month}+u_{t}`$$ (3)

Where *searches* are the total number of Google searches at day *t* (taken in logarithm), while *ϑ*_*day*_ and *ϑ*_*month*_ are, respectively, day and month fixed effects. The final term *u*_*t*_ is the i.i.d. error term. In the second stage, we replace the conflict indicator based on the search query data by its predicted value from the first stage regression.


$$r_{t}=\alpha +\beta r_{t-k}+\gamma x_{t-k}^{j}+\theta \hat{{conflict}_{t}}+\delta_{day}+\delta_{month}+\varepsilon_{t}$$ (4)

The estimation results of the IV approach are reported in columns (4)-(6) of Table 4. Not surprisingly, the first-stage results indicate that our instrument is positively related to the search query intensity. Further, the Cragg-Donald Wald F-statistic to test the strength of the instrument is highly significant and well above the Stock and Yogo [70] weak ID test critical values. The second stage regression confirms our previous findings, as we observe that the coefficient of the predicted value of the conflict indicator remains positive and statistically significant.

Finally, in Table 5, we employ the daily number of casualties of the conflict (in logarithm). This indicator is likely to be more exogenous from an investor perspective. The actions by Hamas and the Israeli armed forces, but also the responses by foreign governments, are taken as given by investors and cannot be directly influenced by them. Once again, when the number of daily casualties increases, investors anticipate that the local conflict might transform into a more regional conflict involving multiple Middle Eastern countries, thereby potentially benefiting the U.S. defense industry.

**Table 5:** U.S. defense ETFs and Israel-Hamas conflict–Casualties.

	iShares		Invesco		Standard & Poor’s	
	(1)		(2)		(3)	
Lagged dependent	0.313	**	0.312	**	0.356	**
	(0.001)		(0.032)		(0.038)	
Casualties	0.013	*	0.016	*	0.015	*
	(0.086)		(0.073)		(0.100)	
Observations	196		196		196	
Pseudo R-squared	0.13		0.09		0.17	
μ_1_	0.21		0.17		0.21	
μ_2_	0.27		0.27		0.36	

Notes: Results of simultaneous estimation of Eqs (1) and (2) using maximum likelihood including the control variables outlined in the main text. Standard errors are heteroskedasticity consistent

**/* Indicating significance levels of respectively 5 and 10 percent.

To summarize the results so far, it appears that investors expect that the Israel-Hamas conflict might, in the short run, escalate into a broader armed conflict, or at least to a real threat to regional peace. This would create a window of opportunity for U.S. defense companies as the arms demand is likely to surge in the Middle East. The empirical results, therefore, provide support for accepting the hypothesis posed above. However, the Middle East is not only an important export market for U.S. defense companies, but also for their European competitors. In particular, three of the top ten arms-importing countries in the world in the past five years are in the Middle East—Saudi Arabia, Qatar, and Egypt. The great majority of arms imports to the Middle East come from the U.S., yet, followed by Europe, especially from France and Italy, constituting about thirty percent. To examine whether investors in the European defense market react in the same way or differently to the conflict, we have re-estimated in Tables 6 and 7 our main models using data from the STOXX Total Market Aerospace & Defense. This mix fund includes only European defense companies (Table A2 in the S1 Appendix shows the top holdings in this mix fund). On the control variables, we used the weighted average of the (long and short-run) interest rates for Germany, Italy, France, and the UK. Besides, we have used the production price index published by the EU/OECD. The results, on average, indicate a much weaker and smaller effect of the risk indicators on European defense companies compared to their U.S. competitors. The explanation for this difference in magnitude is twofold. First, European governments are typically much more reluctant to allow arms exports to the Middle East region. For instance, EU countries regularly reject arms export licenses to this region motivated by regional stability concerns [71]. Besides, European companies are more quickly concerned about the risk of arms embargoes when a conflict escalates [19]. Second, in contrast to the EU, the U.S. put much more foreign policy effort into alliance building in the Middle East. These diplomatic actions typically also involve arms sales.

**Table 6:** European defense companies and Israel-Hamas conflict—Dummy variable.

	STOXX									
	(1)		(2)		(3)		(4)		(5)	
	[0,1]		[0,3]		[0,5]		[0,10]		[0,30]	
Lagged dependent	0.412	**	0.295	**	0.284	**	0.435	**	0.340	**
	(0.012)		(0.018)		(0.006)		(0.013)		(0.012)	
Attack date dummy	0.030	*	0.044	*	0.045	*	0.029		0.020	
	(0.073)		(0.094)		(0.077)		(0.175)		(0.209)	
Observations	186		186		186		186		186	
Pseudo R-squared	0.10		0.14		0.12		0.16		0.14	
Ljung-Box test (p-value)	0.23		0.36		0.34		0.27		0.37	
μ_1_	0.34		0.34		0.11		0.30		0.14	
μ_2_	0.26		0.16		0.16		0.24		0.11	

Notes: Results of simultaneous estimation of Eqs (1) and (2) using maximum likelihood including the control variables outlined in the main text. Standard errors are heteroskedasticity consistent

**/* Indicating significance levels of respectively 5 and 10 percent.

**Table 7:** European defense ETFs and Israel-Hamas conflict–Intensity.

	STOXX					
	(1)		(2)		(3)	
Lagged dependent	0.309	**	0.249	**	0.349	**
	(0.038)		(0.014)		(0.013)	
Google searches	0.017	*	0.012	*		
	(0.076)		(0.089)			
Casualties					0.011	*
					(0.051)	
Estimation method	GARCH		GARCH-IV		GARCH	
Observations	186		186		186	
Pseudo R-squared	0.14		0.18		0.27	
Ljung-Box test (p-value)	0.28		0.38		0.25	
μ_1_	0.24		0.10		0.40	
μ_2_	0.29		0.15		0.19	

Notes: Results of simultaneous estimation of Eqs (1) and (2) using maximum likelihood including the control variables outlined in the main text. Standard errors are heteroskedasticity consistent

**/* Indicating significance levels of respectively 5 and 10 percent.

In the econometric specification, given in Eq (1), we control for the general market return in the U.S. to isolate the impact of the violent conflict. However, when the market return is also affected by this conflict, the results presented so far underestimate the true effect due to endogeneity issues. In that case, there is a general market effect together with a sector-specific effect. To test for this issue, we use the data of an ETF that replicates the yield and return of the S&P500 as our dependent variable. Given the relative insignificance of the share of defense firms in the S&P500 index, the Israel-Hamas conflict is expected not to harm this index. In Table 8, we report the results of this test. Our expectations are mainly confirmed since there is no significant effect of any of the risk indicators on the return of the S&P500.

**Table 8:** S&P500 ETF and Israel-Hamas conflict.

	S&P500 ETF					
	(1)		(2)		(3)	
Lagged dependent	0.309	**	0.350	**	0.243	**
	(0.024)		(0.033)		(0.010)	
Google searches	0.003					
	(0.429)					
Attack date [0,3]			0.012			
			(0.286)			
Casualties					0.006	
					(0.223)	
Observations	196		196		196	
Pseudo R-squared	0.13		0.13		0.11	
Ljung-Box test (p-value)	0.20		0.38		0.30	
μ_1_	0.34		0.21		0.37	
μ_2_	0.33		0.32		0.13	

Notes: Results of simultaneous estimation of Eqs (1) and (2) using maximum likelihood including the control variables outlined in the main text. Standard errors are heteroskedasticity consistent

**/* Indicating significance levels of respectively 5 and 10 percent.

Finally, as argued above, the stock market return of defense-related companies might follow the opposite pattern in the aftermath of a security shock compared to other sectors. Arms-producing companies might benefit from the expected increase in military spending after a security shock, while other industries are more vulnerable to disruptions in the supply chain caused by such a shock. For instance, attacks by Houthi rebels in the Red Sea are jeopardizing trade in a crucial maritime shipping route [72]. To test whether the possible escalation of the Israel-Hamas conflict has a different effect on other business sectors in the U.S., we analyze the impact of the conflict indicators on the ETF returns of various U.S. business sectors, including industrials, health care, utilities, technology, consumer goods, energy, real estate, finance, materials. From the results presented in Table 9, it appears that the effect on other U.S. sectors is limited, as none of the conflict risk indicators has a significant effect on the considered ETFs. Based on this finding, one can conclude that the economic activities of most U.S. sectors are exposed to a limited extent to this conflict and that the U.S. defense industry is a unique sector in this respect.

**Table 9:** The impact of the Hamas-Israel conflict on other U.S. industries.

	Attack dummy	Google searches	Casualties
	(1)	(2)	(3)
Industrials	-0.044	-0.012	-0.011
	(0.217)	(0.493)	(0.293)
Health care	-0.066	-0.014	-0.008
	(0.152)	(0.136)	(0.455)
Utilities	-0.078	-0.026	-0.007
	(0.209)	(0.263)	(0.447)
Technology	-0.084	-0.012	-0.003
	(0.271)	(0.466)	(0.215)
Consumer goods	-0.025	-0.014	-0.005
	(0.211)	(0.327)	(0.121)
Energy	-0.033	-0.015	-0.006
	(0.124)	(0.482)	(0.474)
Real estate	-0.049	-0.007	-0.003
	(0.348)	(0.171)	(0.375)
Finance	-0.026	-0.019	-0.005
	(0.468)	(0.363)	(0.458)
Materials	-0.035	-0.010	-0.008
	(0.351)	(0.397)	(0.182)

Notes: Results of simultaneous estimation of Eqs (1) and (2) using maximum likelihood including the control variables outlined in the main text. Standard errors are heteroskedasticity consistent

**/* Indicating significance levels of respectively 5 and 10 percent.

## 5. Conclusions

On October 7th, 2023, Hamas launched an attack on Israel, initiating a significant military operation in Gaza. Subsequently, a period of uncertainty unfolded, questioning whether the conflict would remain localized or escalate throughout the Middle East. This uncertainty intensified as various regional leaders expressed their concerns or faced accusations of supporting Hamas.

In this study, we aimed to investigate whether investors in the U.S. defense industry expect the internal conflict to become a window of opportunity as it might develop as a real threat to peace in the Middle East. For this purpose, we use different indicators to capture the threat to regional peace caused by the escalation of the internal conflict. These indicators are either based on a structural break after the attack, the intensification of internet searches containing information about the conflict, and the number of casualties caused during this conflict. The findings of this study unequivocally indicate that the equity prices of U.S. defense firms experienced a notable increase, approximately 10–15 percent, in the immediate aftermath of the terrorist attack. This outcome implies that investors foresee the likelihood of the conflict expanding into a more regional event in the near future.

However, one limitation of this study is that, at the time of writing, the Israel-Gaza conflict is still ongoing, although it has not yet escalated into a region-wide conflict. Nevertheless, the scope of the study is to explore the effect of the expectation of escalation or peace threat rather than on any actual escalation.

## Supporting information

S1 Appendix (DOCX)
