# Supplementary material for: The impact of the Hamas-Israel conflict on the U.S. defense industry stock market return
Source: PLoS One. 2025 Feb 4;20(2):e0314677. doi: 10.1371/journal.pone.0314677 (PMC11793771; doi:10.1371/journal.pone.0314677)
Supplement: S1 Appendix — (DOCX) [file pone.0314677.s001.docx]

**Online appendix**

| Table A1: Top ten holdings’ ETFs | | | |
| --- | --- | --- | --- |
| Company | iShare | S&P | Invesco |
| Axon Enterprise Inc |  | 4.00% |  |
| Ball Corp |  |  | 4.05% |
| Boeing Company | 20.40% | 4.58% | 8.20% |
| BWX Technologies Inc |  | 4.15% |  |
| Curtiss-Wright Corp |  | 3.96% |  |
| General Dynamics Corporation | 4.31% |  | 5.34% |
| Harris Corporation | 4.27% |  | 4.06% |
| Heico Corp |  | 3.85% |  |
| Hexcel Corp |  | 4.11% |  |
| Honeywell International Inc |  |  | 6.77% |
| L3 Technologies Inc | 4.29% |  |  |
| Lockheed Martin Corporation | 6.10% |  | 6.92% |
| Mercury Systems Inc |  | 3.76% |  |
| Northrop Grumman Corporation | 4.11% |  | 5.33% |
| Raytheon Company | 4.24% |  | 5.67% |
| Spirit AeroSystems Holdings Inc |  | 4.48% |  |
| Teledyne Technologies Inc |  | 3.81% |  |
| Textron Inc. | 3.17% |  |  |
| TransDigm Group Incorporated | 4.49% | 4.15% | 4.32% |
| United Technologies Corporation | 17.25% |  | 6.75% |

| Table A2: Top ten holdings STOXX Total Market Aerospace & Defense |
| --- |
| Airbus |
| Safran SA |
| BAE Systems |
| Rolls Royce |
| Thales |
| Rheinmetall |
| MTU Aero engines |
| Melrose Industries |
| Leonardo |
| Saab |

*Note: The holding share of this portfolio is dynamic and changes overtime*

|  | Table A3: Correlation matrix | | | | | | | | | | | | | |
| --- | --- | --- | --- | --- | --- | --- | --- | --- | --- | --- | --- | --- | --- | --- |
|  | |  | (1) | (2) | (3) | (4) | (5) | (6) | (7) | (8) | (9) | (10) | (11) | (12) |
| 3 months U.S. T-bill rate | | (1) | 1.00 |  |  |  |  |  |  |  |  |  |  |  |
| 10-year U.S. government bond interest rate | | (2) | 0.47 | 1.00 |  |  |  |  |  |  |  |  |  |  |
| Return S&P 500 | | (3) | 0.02 | 0.04 | 1.00 |  |  |  |  |  |  |  |  |  |
| Change in the Producer Price Index | | (4) | 0.05 | 0.07 | -0.15 | 1.00 |  |  |  |  |  |  |  |  |
| Change in the oil price | | (5) | 0.11 | 0.05 | -0.14 | 0.19 | 1.00 |  |  |  |  |  |  |  |
| Change in real effective exchange rate | | (6) | -0.15 | -0.11 | 0.22 | 0.09 | 0.12 | 1.00 |  |  |  |  |  |  |
| Weapons deal Ukraine dummy | | (7) | 0.10 | 0.18 | 0.25 | 0.16 | 0.17 | 0.22 | 1.00 |  |  |  |  |  |
| Trade volume iShares (in logs) | | (8) | 0.22 | 0.17 | 0.12 | 0.09 | 0.12 | 0.13 | 0.19 | 1.00 |  |  |  |  |
| Trade volume S&P (in logs) | | (9) | 0.21 | 0.22 | 0.23 | 0.06 | 0.09 | 0.21 | 0.21 | 0.89 | 1.00 |  |  |  |
| Trade volume Invesco (in logs) | | (10) | 0.06 | 0.14 | 0.13 | 0.14 | 0.21 | 0.11 | 0.17 | 0.90 | 0.87 | 1.00 |  |  |
| Google searches | | (11) | 0.25 | 0.15 | 0.19 | 0.13 | 0.07 | 0.22 | 0.20 | 0.15 | 0.16 | 0.06 | 1.00 |  |
| Casualties | | (12) | 0.24 | 0.14 | 0.11 | 0.19 | 0.14 | 0.10 | 0.12 | 0.23 | 0.09 | 0.06 | 0.46 | 1.00 |
